# The Advantages and Challenges of Using Endolysins in a Clinical Setting

**DOI:** 10.3390/v13040680

**Published:** 2021-04-15

**Authors:** Ellen Murray, Lorraine A. Draper, R. Paul Ross, Colin Hill

**Affiliations:** 1 School of Microbiology, University College Cork, T12 YT20 Cork, Ireland; 114308696@umail.ucc.ie (E.M.); l.draper@ucc.ie (L.A.D.); p.ross@ucc.ie (R.P.R.); 2 APC Microbiome Ireland, University College Cork, T12 YT20 Cork, Ireland

**Keywords:** endolysin, bacteriophage, antibiotics, multidrug resistance, novel therapy

## Abstract

Antibiotic-resistant pathogens are increasingly more prevalent and problematic. Traditional antibiotics are no longer a viable option for dealing with these multidrug-resistant microbes and so new approaches are needed. Bacteriophage-derived proteins such as endolysins could offer one effective solution. Endolysins are bacteriophage-encoded peptidoglycan hydrolases that act to lyse bacterial cells by targeting their cell’s wall, particularly in Gram-positive bacteria due to their naturally exposed peptidoglycan layer. These lytic enzymes have received much interest from the scientific community in recent years for their specificity, mode of action, potential for engineering, and lack of resistance mechanisms. Over the past decade, a renewed interest in endolysin therapy has led to a number of successful applications. Recombinant endolysins have been shown to be effective against prominent pathogens such as MRSA, *Listeria monocytogenes, Staphylococcus* strains in biofilm formation, and *Pseudomonas aeruginosa*. Endolysins have also been studied in combination with other antimicrobials, giving a synergistic effect. Although endolysin therapy comes with some regulatory and logistical hurdles, the future looks promising, with the emergence of engineered “next-generation” lysins. This review will focus on the likelihood that endolysins will become a viable new antimicrobial therapy and the challenges that may have to be overcome along the way.

## 1. Introduction

Antibiotic resistance presents a growing threat in terms of treating infections. It is a widely recognised problem and the solution will depend at least in part on developing alternative antimicrobials. The World Health Organisation (WHO) has recently referred to antibiotic resistance as “one of the biggest threats to global health, food security and development today” [1]. The WHO has also stated that this ever-emerging resistance will inevitably lead to higher medical costs, longer hospital stays and higher rates of mortality. It is clear that this phenomenon will impact society, including economic, political and social aspects. It is of interest to all in the scientific community to identify and commercialise alternatives to standard antibiotic therapy. Bacteriophage-derived proteins such as endolysins provide one potential solution to this pressing issue. Bacteriophages (phages) have long been used to combat bacterial infections in some parts of the world, but the use of their endolysins is relatively new and is showing great promise. Unlike traditional broad-spectrum antibiotics, endolysins can target specific bacterial taxa with little collateral damage to the surrounding microbiome, and a lack of bacterial resistance mechanisms has also been noted as a positive aspect of phage lysin therapy. These features have resulted in lytic enzymes such as endolysins being assigned to a new class of antimicrobial agents termed “enzybiotics” [2].

Although phage therapy gained some popularity in the 1920s, it was quickly overshadowed by the discovery and development of broad-spectrum antibiotics. The discovery of penicillin in 1928 placed antibiotics centre stage in terms of antibacterial therapies. For many decades, it was believed that broad-spectrum antibiotics were more desirable than a more targeted approach. This idea was further endorsed by pharmaceutical companies as it was much more advantageous to create a product that would be efficacious against multiple infectious pathogens. The era dubbed “The Golden Age of Antibiotics” spanned the 1940s to 1950s and was responsible for the discovery and production of many of the antibiotics still in use today [3]. At the beginning of this period, little was understood about the potential for development of resistant mechanisms in bacteria, and so widespread antibiotic use was not considered to be problematic. It was not until the epidemic of penicillin-resistant *Staphylococcus aureus* in the 1950s that concerns were raised. It was shown that the ability to hydrolyse the beta-lactam ring of penicillin had spread across many different bacteria. To address this issue, methicillin, a synthetic version of penicillin, was introduced. This was followed by the emergence of methicillin-resistant *S. aureus* in the late 1950s [4]. By the 1980s, the discovery and development of new antibiotics had slowed dramatically. Since then, only a handful of novel antibiotics have made it through the commercial pipeline while resistance continues to spread.

**Figure 1:**
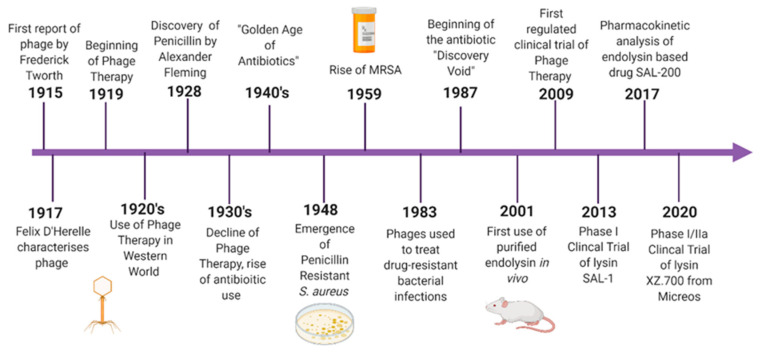
Timeline of Phage Therapy. Outlined is the history of phage and endolysin therapy vs. antibiotic therapy, from the discovery of bacteriophage to the present day.

While the Western world witnessed the rise of antibiotic resistance, phage therapy and research continued to be used, particularly in Eastern Europe. In the mid-1900s, research groups became aware that lysates of phage also possessed the ability to lyse bacterial cells. Studies were carried out to show that this “lytic factor” was capable of killing bacteria even in the absence of the parental phage. These studies were some of the first uses of endolysins as antimicrobial agents. The term lysin was first coined in the 1960s from a study carried out on lysates of *Staphylococcus aureus* [5]. In more recent years, endolysin therapy has become a popular area of research (Figure 1). This involves applying the phage-derived protein exogenously to the bacteria, resulting in cell lysis. Controlled clinical trials of phage therapy have also become more widespread.

### 1.1. Phages

Phages are viruses that can specifically infect and kill bacteria. They were discovered independently by Henry Twort and Felix d’Herelle in 1915 and 1917, respectively. After the discovery of phages, they were quickly put to use against bacterial infections in animals, soon followed by use in humans. In 1919, a phage cocktail was administered to a child suffering from bacterial dysentery and proved to be successful with no side effects [6]. This was the beginning of phage therapy. As a result of a collaboration between French researcher Felix d’Herelle and a team of Georgian scientists led by George Eliava, phage therapy became increasingly popular in the Soviet Union, while it was abandoned in the Western world with the commercialisation of antibiotics [7]. However, in recent years, the emergence of multidrug-resistant bacteria has left the world scrambling for alternative treatments. This worldwide issue could give phage therapy a new lease of life.

Phages are the most ubiquitous and most abundant biological entities on Earth. They are obligate parasites that require a bacterial host to replicate and multiply. They can undergo two life cycles, lytic or lysogenic. The first step is the attachment of the phage to the host bacterium. It achieves this by interacting with receptors on the host cell, followed by tight adsorption onto the cell and the subsequent injection of its genome. The lytic process involves the phage hijacking the host cell machinery in order to produce its viral progeny until lysis of the bacteria releases phage progeny into their immediate environment. Lysogenic or temperate phages form a more long-term relationship with the bacterial host. During lysogeny, the viral genome integrates into the bacterial chromosome as a prophage. At a given stage, usually when the bacterium is in a condition of stress, the prophage can exit lysogeny and begin its replication, lysis and release [8]. The lysis of the bacterial cell that occurs at the end of both replication cycles is made possible by an enzyme called endolysin.

### 1.2. Endolysins

**Figure 2:**
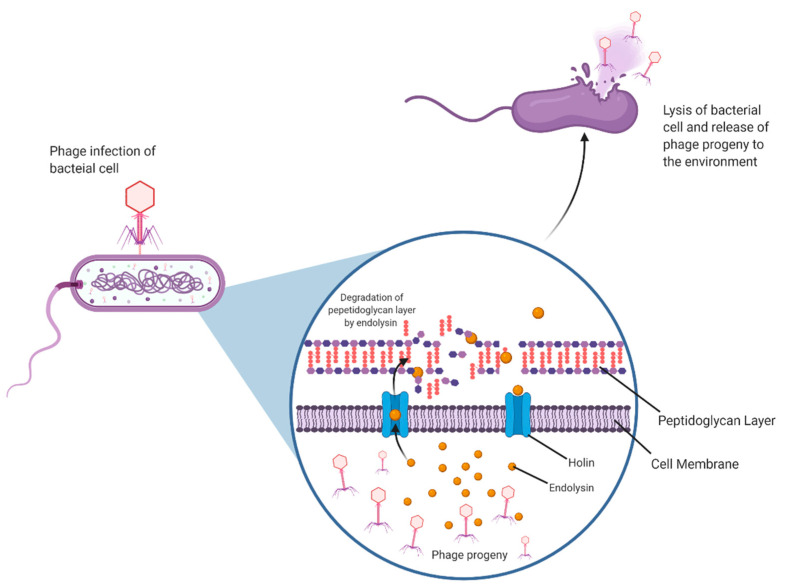
Phage Endolysin Mechanism. The process of a basic phage infection of a Gram-positive bacterial cell, and the role of endolysins and holins in infection.

Endolysins are phage-encoded peptidoglycan hydrolases. These enzymes accumulate within the host cell independently of the phage virion, together with an associated holin protein. As endolysins do not possess their own signal sequences, the purpose of the holin is to allow the endolysin access to the bacterial peptidoglycan by creating pores in the cytoplasmic membrane. This is a highly regulated sequence of events that is only triggered when the holin concentration reaches a certain threshold. Endolysins can now access and degrade peptidoglycan, upsetting the osmotic balance in the cell resulting in lysis and ultimately cell death [6]. Endolysins and holins together are essential to a successful phage infection process (Figure 2).

Endolysins have been of interest as potential antimicrobials because this lytic activity can also occur when the lysin is applied exogenously. They are particularly active in the case of Gram-positive bacteria that do not have a protective outer cell membrane. If administered in the form of recombinant proteins applied externally to the bacterial cells, they can cause rapid lysis and cell death, a desirable trait in an antimicrobial agent. In the case of Gram-negative bacteria, the presence of an outer membrane limits but does not completely prevent the use of endolysin treatment.

Although endolysins have been tested in a number of studies to demonstrate their potential as antimicrobials, there are few cases of these phage-derived proteins actually being put to use in a clinical setting. How is it that such a promising therapeutic has not progressed from the research laboratory to the clinic? Here, we aim to assess the advantages and disadvantages of endolysin therapy and the challenges of implementing endolysins as antimicrobial agents.

## 2. Classification of Phage Lysins

Phage lysins have been reported to largely consist of a two-domain modular structure and are usually greater than 25 kDa in size. The two domains are made up of the enzymatically active catalytic domain and a domain that is responsible for cell wall binding. Lysins of Gram-positive bacteria usually consist of one or more enzymatic domains and a cell wall-binding domain, whereas Gram-negative lysins are often composed of a single catalytic domain. The cell wall-binding domains of endolysins are responsible for their specificity to bacterial targets due to their recognition and binding of ligands noncovalently to a particular substrate in a target cell wall. Therefore, Gram-positive targeted lysins tend to have a more narrow host range, and Gram-negative lysins a more broad range of targets [9].

The bacterial cell wall possesses a mixture of components that are vital for its structure, the balancing of osmotic stress, and regulating what gets in and out of the cell. The cell wall is made up mostly of peptidoglycan (PG). These polysaccharide monomers consist of a disaccharide N-acetylglucosamine (NAG), linked by a short peptide bridge to N-acetylmuramic acid (NAM) residues. The cell wall of bacteria is constantly broken down and remade by a number of enzymes to facilitate processes such as cell division, sacculus expansion during growth, and peptidoglycan remodelling [10].

Endolysins are generally separated into groups based on their cleavage sites. These groups are lysozymes (N-acetylmuramidases), glycosidases (N-acetyl-β-d-glucosamidases), N-acetylmuramoyl-l-alanine amidases, and L-alanoyl-d-glutamate endopeptidases [11]. These enzymes may be placed under the umbrella term of phage-encoded cell wall hydrolases. Endolysins are usually composed of one of these four N-terminals with a variation of a cell wall-binding domain.

Lysozymes (N-acetylmuramidases) kill bacteria through targeted hydrolysis. Peptidoglycan polymers are held together by β-1,4 glycosidic links between the NAG and NAM monomers. Lysozyme acts by hydrolysing these bonds, thus disrupting the structural integrity of the peptidoglycan cell wall [12]. This results in an imbalance of turgor pressure, causing the bacterial cell to lyse. Glycosidases (N-acetyl-β-d-glucosamidases) act to catalyse the hydrolysis of the glycosidic linkage. N-acetylmuramoyl-L-alanine amidases, more commonly referred to as peptidoglycan amidases, act by hydrolysing the amide bond that separates the glycan strand from the stem peptide that lies between the N-acetylmuramic acid and L-alanine residues. L-alanoyl-d-glutamate endopeptidases and interpeptide bridge-specific endopeptidases target the peptide that is composed of the L-Lysine and D-Alanine link [11].

## 3. Why Not Just Use Phages?

Although endolysins have been seen to have promising results in a research setting, one might wonder why a clinician would not just use the parental phage? If the endolysin already exists as a natural part of a phage it might seem counterproductive to clone the protein into a recombinant expression system. The use of genetic modification is a process that will not always find favour, particularly in the food industry, but endolysins have several advantages over phages.

### 3.1. Challenges Facing Pharmaceutical Production of Phage

While the production of most phages in a laboratory setting is a well-established process with reliable protocols in place, the scaling-up of production for industry still poses many challenges. In a lab setting, it is relatively easy to propagate a well-characterised phage for experimental use once the phage, host and selective media are identified. By nature, phages require a bacterial host to replicate. Given that the aim of most commercial companies is usually to eradicate a particular pathogen, it is likely that the host strain required to propagate a phage of interest is pathogenic. Having to grow such strains, many of which are potentially multidrug-resistant, in high volumes to generate a high yield of phage is not a very desirable concept. It introduces an array of safety issues, extra measures to be taken to ensure contamination does not occur, higher costs of production, and a great deal of time. One way of avoiding this issue is using a “surrogate” host instead of the original pathogen [13]. This involves the identification of a non-pathogenic bacteria in the host range of a given phage that would be suitable to facilitate propagation. However, this is not always feasible. Often, phages that are presented as candidates for phage therapy have been isolated and characterised in a research lab. These phages are often praised for having a narrow host range, but this may pose a challenge for the identification of a non-pathogenic host for industrial-scale phage production. This trait that is usually an advantage in the targeted treatment of infection may pose a challenge for the selection of a suitable surrogate host. A non-pathogenic strain that allows the phage to go on to effectively lyse the pathogenic target strain may not always exist [14].

Even in the case of a successful surrogate being available and where large-scale production is possible, a number of disadvantages are still associated with the use of phages in a clinical setting. Several roadblocks stand in the way of such therapy making it to market, and many of these involve regulatory issues. There are still some questions around the use of natural phages and how they will behave in their human host. Although phages are abundant and exist naturally in the human body, the introduction of phages to target a specific bacterium could potentially have long-term off-target effects. Unlike endolysins, the phage virion will interact with the bacterial host and use them for their own replication and potential evolution. It is assumed that the introduction of a phage for therapeutic use will only persist as long as its pathogenic host exists in the environment. In theory, once the infection is eradicated, the phage will not have a host on which to replicate and should pass through the human body. However, phages could potentially mutate and find another target within the microbiome.

### 3.2. Immune Response to Phage Therapy

In recent years, many research groups have investigated the complex role of the immune system in phage therapy and these studies have revealed that phages interact with mammalian cells. The body of work surrounding this topic largely indicates that the interaction between phages and the host immune system does not have a significant impact on the success of phage therapy, but this theory is conflicted. Some studies have investigated whether the immune system could be responsible for the clearance of phages administered during therapy, as anti-phage antibodies have been reported to be present in humans. A 2016 study carried out in patients undergoing experimental phage therapy investigated the humoral response to this treatment by examining the release of IgG, IgA, and IgM anti-phage antibodies [15]. The phage therapy involved the administration of an MS-1 phage cocktail to target Staphylococcal infections. The cocktail was administered orally or locally depending on the patient. The study showed that out of 20 patients, the majority receiving the treatment did not display an increase in anti-phage antibodies in their sera. Some individual cases arose where there was an increased level of IgG and IgM antibodies, but this did not affect the overall success of the treatment. On the other hand, patients who were recorded to have anti-phage antibodies present in serum before the administration of MS-1 (23% of patients) did not respond as well to the treatment. The authors state that the results of the study suggest that phages can induce the production of anti-phage antibodies, but this depends on several factors. It is suggested that the duration of treatment, phage dose, and route of administration influence the immune response, alongside the immune status of the individual patient prior to treatment. Overall, there is a limited bank of data suggesting that the immune system could negatively affect the outcome of phage therapy. Reports indicate that the response is often case-specific to the phage being used and to the patient’s own immune system. To conclude whether this is a viable challenge to the future of phage therapy a larger volume of data needs to be generated from controlled clinical trials.

### 3.3. Implications of Horizontal Gene Transfer

A number of phages have also been identified that can facilitate horizontal gene transfer. One of the most well-characterised phage that has played a role in this gene transfer is the temperate phage CTX, which targets *Vibrio cholerae*. CTX is a filamentous, single-stranded DNA phage. It is widely reported that the CTX prophage is responsible for the presence of genes encoding the cholera toxin ctxAB. Strains that are capable of producing this toxin, such as *V. cholerae* O1 El Tor and *V. cholerae* O139, are more pathogenic than their predecessors as this toxin is the causative agent of diarrhoea – which is a classical feature of cholera infection. In the late 1990s, it was discovered that the genes for the notorious cholera toxin were encoded in the genome of CTXφ [16]. The genome of CTXφ is split into two parts—the RS2 sequence and the core region. The RS2 region is responsible for prophage replication and integration. The core region consists of numerous genes associated with phage assembly and structural formation, but also includes elements zot and ace that code for cholera toxin subunits. Once the phage carrying these elements infects susceptible *V. cholerae* cells they integrate their DNA into the chromosome of *V. cholerae* at the attB (dif) integration site [17]. This process facilitates the horizontal gene transfer of toxin ctxAB. The ability of phage to cause this type of new toxicity in bacteria is a clear cause for concern and places doubt over their safety profile as a therapeutic agent.

The emergence of this cholera toxin is but one example of the potential for horizontal gene transfer mediated by temperate phages. The mobilisation of genetic elements by prophages could not only give rise to increased toxicity and virulence of pathogens but can also include the transfer of antibiotic resistance genes [18]. For phage therapy to be a viable treatment option, temperate phages should not be used. The administration of a temperate phage to large cohorts of patients could have negative effects on the general population. Instead of curing an infection, they could have the opposite effect, increasing the pathogenicity of the bacteria.

### 3.4. Phage Resistance Mechanisms

It has also been reported that phages themselves may succumb to resistance mechanisms. The ability of bacteria to become resistant against phages has been recognised for a long time. A 2007 study shed light on one of the mechanisms that allow resistance to occur. This report involved the bacterium *Streptococcus thermophilus* and focused on clustered regularly interspaced short palindromic repeats (CRISPR)-mediated resistance to phage infection. The authors showed that there was a correlation between the difference in spacers in phage-resistant and phage-sensitive mutants of *S. thermophilus*. The addition of new CRISPR spacer units was associated with the development of resistance against phage. It was deduced that these spacers were derived from the incoming phage DNA and that if the spacers were a perfect match to the phage DNA, the bacteria were resistant to that phage. Any discrepancy in the sequence meant the bacteria remained sensitive to phage infection. The study also showed that the removal of CRISPR spacers could reverse the resistance phenotype [19].

A more common means of host resistance to phages can occur through receptor modification. Phage infection is initiated through adsorption, where the phage recognises and binds receptors on the bacterial cell surface. Bacteria have evolved to prevent the attachment of phage by modifying these receptors. Resistance mechanisms that act by blocking phage adsorption are reported to fall into three categories. The first route is the blocking of phage receptors. This involves the target bacteria modifying the structure of their cell surface or modulating their three-dimensional conformation. As a result, the proteins responsible for host recognition are rendered useless and the phage can no longer gain access to the bacterial cell. The second means of achieving resistance to phage adsorption is the production of an extracellular matrix. The production of extracellular polymers creates a physical barrier between the phages and their receptors. However, some phages can overcome this mechanism by recognising and degrading these barriers through the action of hydrolases and lyases. The final means of combating adsorption is the production of competitive inhibitors. These inhibitors are molecules that would normally be found in the bacterial environment but can bind to the phage receptors. If the phage receptors are already bound, it is impossible for them to freely bind to the bacterial cell receptors, thus interrupting phage infection [20].

Another class of resistance mechanism adopted by bacteria are restriction-modification systems. Restriction modification is considered to be a natural defence system in bacteria that allows them to recognise foreign DNA entering the cell, for example, phage DNA, and cleave it. This cleavage is carried out by restriction endonucleases recognising and cleaving short DNA sequences in the cell—these are called restriction sites. A second component called methyltransferase protects the host DNA by methylating it and as a result, the host DNA is unaffected. Some phages have also evolved to evade this defence system by reducing the number of restriction sites in their own genomes [21].

## 4. Recent Endolysin Success Stories

Endolysin therapy usually works best with Gram-positive bacteria, due to their natural structure of exposed peptidoglycan cell walls. When applying endolysins exogenously, lysis occurs without the need for holins or other partner enzymes. This section will discuss just a few of the many Gram-positive, and Gram-negative bacteria that have become targets for endolysin therapy.

### 4.1. Gram-Positive Targets

*Staphylococcus aureus* is a Gram-positive bacterium that has been the focus of several endolysin studies in recent years. Multidrug-resistant *S. aureus* (MRSA) is a superbug that can cause infection in a variety of sites on the human body and is often acquired in a hospital setting. These nosocomial infections are often difficult to treat and have often been acquired by an individual who is already ill. As treatment of staphylococcal infections with antibiotics is often unsuccessful, endolysin therapy shows promise to tackle this bacterium. A 2010 study showed that the phage-derived lysin MV-L had a significant effect against MRSA [22]. MV-L is encoded by the phage φMRII. MV-L was shown to have a rapid effect against MRSA, with the complete killing of the bacteria achieved in as little as 15 min. It was also shown to have a narrow host range, allowing it to be potentially used against MRSA in a complex community of bacteria. After initial analyses, MV-L was brought forward to be tested in vivo. The nasal passages of mice were inoculated with MRSA, and the bacteria was left to establish for 60 h. MV-L or a buffer control were then administered intranasally to the mice. In the control group, visible colonies appeared in the samples of nasal tissue after 6 h—an average of 1818 CFU/nasal cavity. In contrast, mice treated with MV-L only had an average of 61 CFU/nasal cavity. Furthermore, 1 out of 9 mice displayed complete elimination of infection after treatment with MV-L. The study went on to administer a lethal dose of MRSA to the mice by intraperitoneal injection. This led to the septic death of all mice within 24 h. However, when MV-L was administered intraperitoneally 60 min after infection, a lifesaving effect was observed.

The food industry is another area that has an issue with *Staphylococcus* contamination, particularly in terms of biofilm formation. A 2019 study looked at using endolysins as an alternative to eliminate *S. aureus* biofilms that form on food contact surfaces. The phage-derived recombinant lysin “LysCSA13”, produced by bacteriophage CSA13 was cloned into an *Escherichia coli* expression system. The resulting protein was used against staphylococcal strains on several common surfaces, including polystyrene, glass, and stainless steel. LysCSA13 demonstrated strong lytic activity and reduced the biofilm mass by 80–90% across all conditions. Treatment resulted in up to a 3-log unit reduction compared to the untreated control [23]. An added advantage to using endolysins as a biocontrol agent in the food industry and food production environments is their specificity to bacterial cells. The use of such antimicrobials would not affect their human host and little to no effect on non-target bacteria.

Efforts have also been turned to targeting spore-forming Gram-positive bacteria such as *Bacillus cereus* and *Clostridium* with phage-derived lysins. *B. cereus* and *Bacillus anthracis* are pathogenic bacilli that can cause serious harm to their hosts in the form of food poisoning and anthrax, respectively. Phages were historically used as an indicative test for anthrax poisoning due to their high specificity to the bacterium. The *Clostridium* genus includes a number of significant human pathogens including the bacteria responsible for botulism and tetanus, along with the hospital-acquired gut infection due to *Clostridioides difficile* [24].

A 2012 study used an endolysin designated LysB4 that was isolated from the *B. cereus* phage B4. This lysin was characterised as an endopeptidase with a two-domain structure, cleaving the peptidoglycan L-Ala and D-Glu bonds. This endolysin had strong lytic activity against *B. cereus* over a wide range of environmental conditions. It was important to address each of these parameters due to the notorious ability of spore-formers to survive in environmental extremes. Strong lytic activity was observed at pH 8.0–10.0, and the lysin remained active at pH 2.0–10.5 over 30 min incubation. A range of temperatures was also examined from 37 to 75 °C, and the optimal temperature was 50 °C. However, the protein was inactivated at 55 °C and above. NaCl concentrations were also tested, with the activity of LysB4 reducing as NaCl concentrations increased, resulting in an approximate 60% decrease in activity at 200 mM NaCl. The performance of LysB4 in each of these tests suggests it is a promising candidate for the biocontrol of spore-forming bacteria [25].

An endolysin has been cloned from the *Clostridium* bacteriophage CD27. This endolysin, cd27l, was initially tested against *C. difficile* cells and showed promising lytic activity. It was subsequently tested against a total of 30 *C. difficile* strains and killed all strains examined. A significant finding of this study was that even though the endolysin is active against *C. difficile*, it showed little to no activity against *Clostridium*-like species that are commensal to the human gut microbiome. This observation means that phage-derived therapy for *C. difficile* infections could be used without risking collateral damage to the surrounding community. This is a property that cannot be matched by the antibiotics that have typically been used to treat these kinds of infections [26]. However, several challenges such as delivery to the target site remain an issue for endolysin therapy, especially in such a complex environment as the human gut.

Other studies have also focused on the potential of endolysins to aid or replace antibiotics in the food production chain. *Listeria monocytogenes* is a problematic pathogen in the production of many foodstuffs such as processed meats and dairy products, particularly soft cheese. PlyP100 is a recombinant endolysin that has been engineered to cope with the environmental conditions in which *Listeria* is often found. These include extremes of heat (pasteurisation and heat-treatment steps), high NaCl concentrations, and low pH. In addition, it was shown that PlyP100 could continue to act on *L*. *monocytogenes* for up to four weeks in refrigerated storage [27]. This antimicrobial protein shows considerable promise as a specialised, cost-effective, and highly efficient method of biocontrol in terms of food processing, shelf life, and storage capacity.

### 4.2. Gram-Negative Targets

Gram-negative endolysin therapy is not straightforward. Due to the presence of a protective outer membrane, phage lysins do not have a naturally exposed target. The outer membrane is composed of phospholipids and lipopolysaccharides. The lipopolysaccharide molecules are held together by the phosphate bonds present between the acidic sugars. Researchers have been creative in finding a way to get past this hurdle and allow the endolysins to freely access the peptidoglycan cell wall. Pre-treatments have been utilised to overcome the cell membrane barrier. One such method is using EDTA to permeabilise the Gram-negative cell membrane. EDTA is a chelating agent that removes outer membrane-stabilising divalent cations from their binding site, disrupting the membrane and leaving it vulnerable to lysin attack [28]. Permeabilising the outer membrane may also be carried out mechanically. High hydrostatic pressure (HPP) is an alternative method of food preservation that can inactivate some microorganisms and enzymes. HPP has been used to make Gram-negative bacteria sensitive to molecules such as bacteriocins and antimicrobial peptides by optimising parameters such as time and pressure [29].

An alternative approach to targeting Gram-negative bacteria is the fusion of endolysins to components that allow them to gain access to the peptidoglycan layer. One such example is the fusion of the *E. coli* phage lysin lysep3 with the *Bacillus amyloliquefaciens* endolysin-binding domain D8. Lysep3 alone does not possess the catalytic ability to breach the outer membrane of *E. coli*. However, when engineered to fuse with the D8 cell wall-binding domain, it can lyse the bacteria from the outside. The D8 domain has the ability to disrupt the integrity of the outer membrane of the bacteria, thus allowing the enzyme portion to reach the target peptidoglycan membrane [30].

*Pseudomonas aeruginosa* is a bacterium that is known for its resistance to antibiotics, making it a clear candidate for endolysin therapy. It is often a hospital-acquired infection that can be responsible for pneumonia, urinary tract infections, and bacteraemia. In 2017, a phage-derived endolysin LysPA26 was isolated with lytic activity against a multidrug-resistant strain of *P. aeruginosa* without the use of an outer-membrane permeabiliser. The study published impressive results showing the lysozyme decreased bacterial numbers by up to four log units in 30 min. It was also shown to be pH and heat-stable. Furthermore, the endolysin had a broad range of activity, also having an effect against other Gram-negative bacteria including *Klebsiella pneumonia*, *Acinetobacter baumannii,* and *E. coli*, all of which are known to be multidrug-resistant [31]. LysPA26 is no doubt a strong candidate for the future of endolysin therapy. Being able to selectively target Gram-negative bacteria in such an effective manner without the use of antibiotics, as is shown in this study, is indeed possible and could be a first-choice therapy in the future.

### 4.3. Targeting Biofilms

Bacterial biofilms are notoriously difficult to eradicate. Biofilms form when bacteria attach to a surface and grow as a connected community of cells. This layered formation means that only a certain number of bacteria on the outer layer of the biofilm are vulnerable to antimicrobial agents while the bacteria within are protected and so can continue to proliferate. This often establishes a chronic infection resulting in long-term antibiotic use. Due to the nature of this treatment, it is common for biofilm-forming species of bacteria to become resistant to the antibiotics used. Endolysins have recently shown promise as a remedy to these recalcitrant infections.

When *Staphylococcus* strains cause infection in the form of a biofilm they are often difficult to treat because many of these strains are already multidrug-resistant. In 2018, endolysin LysGH15 was shown to have antimicrobial effects against a number of these strains. In this study, *S. aureus*, *S. epidermidis*, *S. haemolyticus,* and *S. hominis* were tested both in vitro and in a mouse model. In the in vitro studies, all *Staphylococcus* strains showed sensitivity to LysGH15 with up to a four-log reduction in bacterial counts in as little as 30 min at a concentration of 20 µg/mL. In biofilm formation studies, a dose of 50 µg/mL proved to be effective. Established biofilms of various *Staphylococcus* species were also investigated. In this instance, biofilms were allowed to form for up to 72 h and were treated with 100 and 150 µg/mL of LysGH15. At the concentration of 150 µg/mL, levels of some established biofilms were destroyed by the lysin. Other strains showed a lesser, but still significant, reduction of 51–81% of the biofilm versus PBS controls.

In vivo studies were performed to investigate the potential of LysGH15 to protect mice from *S. epidermidis* infections. Mice were first injected intraperitoneally with a lethal dose of *S. epidermidis*. One hour post-infection, LysGH15 was administered by intraperitoneal injection at either 5, 20, or 20 µg/mL. All mice treated with the PBS control died within 48 h, but the mice given the endolysin made a significant recovery with a decrease in bacteraemia. LysGH15 was also tested for its potential to induce resistance in the bacteria strains used during this study. This was carried out on both vancomycin-resistant and vancomycin-sensitive strains of *S. epidermidis*. It was reported that no spontaneous resistant mutants were recovered and cells collected from each generation of sub-cultured cells retained their sensitivity to the lysin [32]. This study highlighted many favourable traits for endolysins against biofilms, including the ability to penetrate the core of the biofilm formation and a lack of resistance emerging against the lysin.

### 4.4. Synergy

Although there are many cases of individual endolysins showing success as antimicrobials, there have also been instances where more than one lysin has been used together, or a lysin was combined with another class of antimicrobial, to obtain a synergistic effect against infection.

In 2010, a study was published that showed the synergy between a chimeric lysin and a traditional antibiotic against methicillin-resistant *S. aureus*. The chimeric lysin ClyS was formed through the fusion of the catalytic domain of phage lysin phiNM3 with the cell wall-binding domain of another *S. aureus* endolysin. The resulting lysin was shown to act against methicillin-resistant, vancomycin-intermediate resistant, and sensitive strains of *S. aureus* in a mouse model. However, the study went on to investigate the combination of this engineered phage-derived lysin and oxacillin. In vivo experiments showed that in an MRSA septicaemia model, mice were administered bacterial suspensions through a modified method of the intranasal colonisation model. The control group had a 13% survival rate. In individual treatment groups, the survival rate was between 30 and 35%. However, when lysin ClyS was combined with oxacillin the survival rate was raised significantly to 80–82% [33].

Cases like these show that in some cases antibiotics do not need to be replaced entirely but can be paired with molecules like lysin to make them more effective at lower doses. This could give traditional antibiotics a second lease of life.

## 5. Safety, Pharmacokinetics, and Pharmacodynamics

Although a lot of research is being performed on the efficacy of endolysins against bacterial targets, not a lot of literature has been published that focuses on their safety profile. Likewise, data surrounding the pharmacokinetics and pharmacodynamics of endolysins as potential drugs is limited. Pharmacokinetics can be described as what the body does to a particular drug, whereas pharmacodynamics looks at what effects the drug has on the body. All of these factors are important aspects of getting a drug approved for therapeutic use. Here, we will analyse some available data on the interaction between endolysins and the human host.

A 2018 study looked at the safety of two endolysins that target *Streptococcus pneumoniae* [34]. Endolysins PaI and CpI-1 were tested for their toxicity and preclinical safety. Immune monitoring was carried out on human macrophage and pharyngeal cells. Once endolysin was added to the cell lines, changes in gene expression were monitored over the following six hours. This study also covered in vivo toxicity by monitoring IgG levels in mice exposed to lysin over 30 days.

The study found that there were no notable changes in inflammatory gene expression, cellular and non-cellular immune responses, or microbiome changes in the mammalian cells or systemic function. In the mouse studies, IgG levels did rise. However, this effect was expected due to the proteinaceous nature of lysins. Despite this response, the immune cells did not have a negative effect on the catalytic ability of the endolysins. Overall, it was shown that endolysins PaI and CpI-1 do not present any significant levels of toxicity. Studies like these are important to create a body of literature around the safety of endolysins and their suitability for clinical use.

The lysin MV-L has been previously mentioned for its activity against MRSA. However, the authors of this paper also looked at the host immune reaction to the use of this lysin [22]. Six mice were used in this study and were injected intraperitoneally at a dose of 500 U with the lysin. Western Blot analysis detected antibodies reactive to MV-L in the serum of all six mice, in contrast to the control group that had no detectable antibodies present. ELISA analysis also showed a significant level of antibodies present in comparison to the control. Although levels of a humoral immune response were detected in the mice, they did not appear to interfere with the efficacy of the endolysin treatment. The authors also noted that the mice were administered a single excess dose of lysin (1500 and 2000 U), followed by a repeating dose of 2000 U, with neither scenarios revealing any visible adverse effects in the mice, nor did it affect their survival rate.

Preclinical safety evaluation of the recombinant endolysin SAL-1 (as part of SAL200) was carried out in 2014 [35]. This study looked for signs of toxicity in two animal models—rodents and canines. SAL200 was administered intravenously to both groups in single or repeated weight-dependent doses. Safety pharmacology tests were also carried out which included assessment of the effect of the lysin on cardiovascular, respiratory, and central nervous functions. Immunogenicity was also assessed by the analysis of anti-SAL-1 antibody and C3 complement levels in the blood.

Overall, no signs of toxicity were observed in the rodent single- or repeated-dose tests for central nervous system function of respiratory function. Some adverse effects were recorded among the canine group who received multiple doses of SAL200 for more than a duration of 1 week. However, it is noted that these effects were mild and self-limiting. The authors state that these effects were likely the result of an immune response to the anti-SAL-1 antibody production, activating the host’s complement system. Going forward it is suggested that a clinical study design could be adapted to avoid this mild adverse response to the treatment, for example, giving doses over a shorter period. The safety evaluation as a whole supported the progression of the treatment to clinical trials. The outcome of recent clinical trials will be discussed later in this review.

Although the results of safety evaluations and immunogenicity studies to date largely support the idea of endolysins as a potential therapeutic, a greater volume of research in this area needs to be carried out to ensure that different classes of lysins do not pose a significant risk to the host.

### Characteristics of Endolysins Versus Antibiotics

As previously mentioned, endolysin therapy is a relatively new concept and as a result, the amount of data available about their behaviour as pharmaceutical drugs is limited. However, antibiotics have been widely used for decades and so their pharmacokinetics, pharmacodynamics, and toxicity are well documented. Antibiotics combat infections through inhibition of cell wall synthesis, DNA replication, or protein synthesis. They do not have a direct effect on innate immune cells but may cause the release of pathogen-associated molecular patterns (PAMPs) as a result of bacterial cell lysis and are not reported to induce the formation of antibodies [36]. As mentioned, endolysins do interact with the host immune system and cause induction of antibodies, which would generally be expected of a protein-based drug, but studies to date have not indicated that this would have a negative impact on the recipient of the treatment, nor significantly affect the efficacy of the treatment itself. Similar to antibiotics, endolysins result in lysis of bacterial cells and so the release of PAMPs would also be expected here.

One clear advantage that endolysins have over their traditional counterparts is host specificity. The majority of antibiotics were pursued as therapeutics due to their broad-spectrum mode of action—one antibiotic could be approved and manufactured to be prescribed for several illnesses. One significant issue is the effects that this has on the human body by means of the collateral damage that occurs within the microbiome. It is often the case that when an antibiotic is killing a pathogen, it also wipes out healthy commensals. These off-target effects are capable of significantly disrupting the microbiome, sending it into dysbiosis. This has been linked to a number of health implications including metabolic disorders, diabetes, malnutrition, and *Clostridium difficile* infection [37]. The targeted action of endolysins allows for a single pathogen to be singled out from a cohort of bacteria, and avoids the needless killing of commensals.

## 6. Limitations of Endolysin Therapy

Although there are many positives to the use of phage-derived lysins in a clinical setting, their widespread use is not without challenges.

### 6.1. Drug Delivery Methods

Although there are a handful of endolysin products on the market, there is a common theme in their method of delivery in that the vast majority are applied topically. Ideal in cases such as skin infections, but topical application of lysins is not suitable for many parts of the body, for instance in treating gut infections.

The most common route for gut-targeted therapies, and certainly the most popular amongst patients, is the oral delivery route. The majority of drugs on the market are either in tablet or liquid form and are usually taken orally. However, this is not without difficulty. Given that drugs in this form must transit the stomach and digestive system, they are immediately vulnerable to an arsenal of enzymes, different pH levels, and mechanical digestion. These can all affect the integrity of the drug molecule and the bioavailability of the drug systemically.

As endolysins are proteinaceous, they can quickly be affected by these processes, rendering them useless. Stomach acid can disrupt the structure of certain endolysins. Proteases such as trypsin, chymotrypsin, pepsin, and peptidase act by degrading proteins. Many endolysins possess cleavage sites that can be targeted by these enzymes [38]. In some instances, the oral route of delivery is not always suitable, for example, targeted delivery to the lung. In recent years, several viable lysins have been isolated to combat lung pathogens but getting them to the site of infection effectively is not a straightforward task. Similar to the human gut, the lungs also have many immune cells and enzymes that would quickly target proteins and peptide drugs. Lysins would likely be degraded by proteases or alveolar macrophages [39].

In the literature, endolysin treatment is most commonly given to human subjects in clinical trials as intravenous (IV) injection. This allows the drug to be administered straight to the bloodstream and circulated systemically, thus avoiding the problems posed by the digestive system during oral delivery. However, IV as a route for drug delivery is not without its challenges. IV drug delivery is much more invasive than the traditional oral route and requires the patient to attend a specialised clinic which may be time-consuming and expensive. The injection site of intravenous therapy also poses its own potential complications leaving the patient at risk of a secondary bacterial infection at the injection site [40]. If endolysin therapy is to become commonplace, such factors would certainly have an impact on patient perception and uptake. The general public would almost certainly favour a traditional antibiotic taken orally, rather than a new therapy with a more invasive delivery route.

Another route for drug delivery that may be considered is the nasal route. This method of drug delivery is relatively non-invasive and patient-friendly. However, this too has its own limitations. The nasal cavity is small and therefore has a limited surface area of approximately 150 cm^2^ for the drug to use for its adsorption, a fraction of the size of the gastrointestinal tract. This may affect the bioavailability of the endolysin at the target site. There may also be an issue with the size of the protein of interest, and whether it would be too large to cross the nasal mucosa. A study on the delivery of high-molecular-weight drugs in 2009 indicated that proteins over 1000 Da in size would encounter difficulty crossing the nasal epithelia [41]. Given that endolysins on average range from 15 to 40 kDa in size, this may impact their suitability to be administered through the nasal route.

To avoid these natural defence systems, endolysins would have to be further engineered. Recently, there have been several studies investigating ways to protect endolysins from the host’s immune system. An example of this would be encapsulating the protein in tiny structures that allow for its timed-release at the site of infection. The release of the endolysin can be designed to occur at a certain temperature or a specific pH. These options will be explored further in Section 7 of this review.

### 6.2. Not A “One Size Fits All” Concept

Although a benefit of endolysin and phage therapy is their specificity, there may be a practical issue that is overlooked in the scramble to find an alternative to antibiotics. Of course, this targeted approach is of vital importance in a world where antibiotic resistance is common, especially when scientists were not ready for the emergence of resistance to the popular broad-spectrum category of antibiotics.

In an ideal world, endolysin/phage-related therapy in general would be sufficiently broad spectrum that a patient in a doctor’s office could describe their symptoms, and a certain phage or a standard cocktail would be prescribed. In specialised settings, lysins have shown positive results on specific infections, many of them serious cases. However, the majority of antibiotics are prescribed post-operatively in hospitals or are used by members of the public who have simple illnesses such as tonsilitis, pulmonary and otic infections. Is it practical to have such a targeted and personalised approach to these common illnesses? How can we bridge the gap between the use of a precise phage cocktail given to a patient with an already identified pathogen and the use of antibiotics in an individual with an unidentified infection? Personalised medicine is constantly evolving and hopefully in years to come will be the gold standard of practice. However, although it has a bright future, it is still in its infancy and it is unknown when it will become common practice.

### 6.3. Regulatory Body Approval

In Europe, the European Medical Agency (EMA) is the regulatory authority responsible for deciding the fate of new drugs and therapeutics. This is made up of 50 regulatory authorities across 31 countries. This committee oversees many aspects of a new drug such as its manufacture and production, marketing, clinical trials, and safety monitoring [42]. Endolysins are most commonly produced through recombinant DNA technology. This method of expressing the protein of interest in a host vector, such as *E. coli*, has a specific set of requirements if they are to meet EMA guidelines. Many endolysins showing promise are genetically engineered to have a specific action, and so face more hurdles on the road to production and use in clinical settings. The requirements are listed under 2001/83/EC and EC regulation 726/2004.

Currently, there is only one endolysin-based treatment that has made it through EU restrictions for human use and has been accepted as a medical product. Lysin SA.100, otherwise known as Gladskin, is produced by Micreos. Given the amount of research and successful isolation of functional endolysins with viable medical applications, this success rate is extremely low. However, there may be more hope for veterinary-based endolysin products as the restrictions outlined for animal use allow endolysins to treat certain infections [43].

## 7. Looking to the Future—Next-Generation Lysins

**Figure 3:**
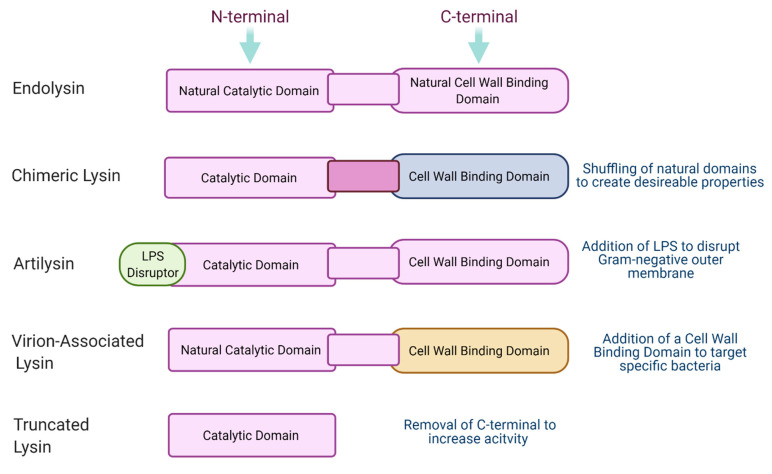
Next-Generation Lysins. This illustration shows the basic difference in structure between the following: natural endolysins, the engineered chimeric lysin which involves the linking of the catalytic domain of one lysin to a suitable cell wall-binding domain, the Artilysin which involves the fusion of an LPS disrupting molecule to a lysin, VALs which do not possess their own cell wall-binding domain and so are fused to a suitable candidate, and truncated lysins which have the cell wall-binding domain removed to increase activity.

In recent years, a new generation of phage-derived lysins have been proposed. These new and improved lysins may allow us to overcome existing hurdles and make phage lysin therapy more accessible. Modifications of endolysins allow researchers to create an “ideal” lysin that maintains high activity while possessing other desirable traits depending on the circumstances of its use. An outline of these modifications can be seen in Figure 3. An ideal lysin might have properties such as thermostability, high solubility to make it bioavailable, may be highly targeted or broad spectrum depending on the target infection, may be protease-resistant if being used in the gut, and should be economical to produce. Engineering, and specifically designing, endolysins gives countless opportunities for a popular, commercially used product to be produced.

### 7.1. Virion-Associated Lysins

Virion-associated peptidoglycan hydrolases (VAPGH) or virion-associated lysins (VAL) are another class of phage-encoded proteins that have antimicrobial properties. These are tail-associated muralytic enzymes associated with the structure of the phage tail and are usually termed exolysins. These proteins are active at the initial stages of infection during adsorption, allowing the phage tail to penetrate the bacterial cell wall. They are found to act specifically on the cell wall of viable and dividing bacteria with a glycosidase or endopeptidase mode of action. These proteins have also been shown to be promising as they have a dual catalytic domain which reduces the risk of the emergence of resistance.

In recent years, VALs have been engineered to target certain bacteria by fusing them into a chimeric lysin. A 2015 study used a chimeric lysin EC300 to target *E. faecalis* during its growth phase [44]. EC300 is a hybrid of an endopeptidase domain of a VAL and a cell wall-binding domain of an amidase endolysin. The study showed the engineered chimeric lysin to have a clear advantage over each of its parts alone, and even over commercially available antibiotics. An earlier report also illustrated the therapeutic potential of phage-encoded chimeric proteins [45]. This study involved the joining of *S. aureus* endolysin LysH5 with two fusion proteins between lysostaphin and a virion-associated lysin HydH5. When this hybrid protein was tested against *S. aureus* over time, no resistance to the antimicrobial was detected even after ten cycles of bacterial exposure to the phage lysins. The proven efficacy, together with low resistance development, highlights the potential for virion-associated lysins as an alternative therapy to bacterial infection.

### 7.2. Chimeric Lysins

Chimeric lysins (chimeolysins) are an engineered form of natural phage endolysins often formed by the shuffling of the catalytic N-terminal domains and cell wall-binding domains. This rearrangement of the lysin can give rise to enhanced activity, can make the lysin more soluble, or can confer a wider host range. As previously mentioned, artificial lysins have been successful in making it to commercial use with products like Gladskin receiving large investments and breaking into the US market. The development of chimeolysins gives a new lease of life to endolysins, allowing them to leave the lab and translate to clinical applications.

A recent study looked at the ability of a phage-derived endolysin HY-133 to tackle *S. aureus* biofilm formation in vascular graft infections [46]. These infections result from biofilm colonisation of prosthetics following surgery and are reported to contain bacteria with 1000-fold greater resistance to antibiotics. The endolysin was tested alongside existing antibiotics daptomycin and rifampin. HY-133 is a recombinant chimeric lysin formed from the N-terminal domain of phage K and the cell wall-binding domain from lysostaphin. The study concluded that daptomycin has the strongest effect on the biofilm, but HY-133 has a moderate effect on the graft surface, especially after an incubation time of 18 h. Rifampicin was not effective as an antimicrobial for this biofilm. As the use of antibiotics for the treatment of bacterial infections could pose a risk of resistance, it could be said that the endolysin would be the most favourable antimicrobial agent in this setting.

ClyF is an example of a chimeric lysin that has been engineered specifically to target MRSA. ClyF is derived from the natural endolysin Pc but was modified by the formation of a hydrophobic cleft in its N-terminal and an immunoglobulin-like structure in the cell wall-binding domain. Natural *S. aureus* phage lysins have been reported to display issues with solubility and decreased activity that would greatly hinder antimicrobial action, particularly against biofilms. This lysin was chosen via a lysis-based rapid screening method to identify chimeolysins that the research group designed in a previous study. This screening method involved expressing a chimeolysin library constructed from donor catalytic domains and cell wall-binding domains in an *E. coli* expression system. This system was combined with a novel enzyme ClyN, which when released allows for the detection of lytic activity against desired bacterial targets [47]. Once identified, ClyF underwent several assessments to test its suitability to act against multidrug-resistant *S. aureus* in both sessile and planktonic states. ClyF showed an increase in thermostability and a greater range of tolerance to pH. ClyF was used against bacteraemia and wound infection models in mice and showed good efficacy against planktonic and biofilm MRSA [48].

Another research group has proposed a lysin modification system to increase their efficacy. It has been shown that the addition of cysteine to the end of the C-terminal of antimicrobial peptides such as lysins can increase their antimicrobial activity. The modification template used for this study was called CTC modification strategy (cysteine to its C-terminus). It was reported that the addition of this additional cysteine can improve the efficacy of lysins against both Gram-positive and Gram-negative pathogens and may be used regardless of the original lysin sequence. Although the exact mechanism of this approach has yet to be defined and results can vary, the increase in antimicrobial action has been shown to be at least two-fold [49].

*Acinetobacter baumannii* is another pathogen that has been targeted by a new generation of engineered lytic enzymes derived from phages. A 2016 study saw the modification of *A. baumannii* lysin PlyF307 to create the optimised P307SQ-8C [50]. A sequence of amino acids present in PlyF307 was identified as the full cell wall-binding domain of the lysin. This is P307, consisting of eight amino acids that constitute the full native C-terminal domain for this endolysin. These eight amino acids were fused to a further eight (SQSREQC) to form the final molecule P307SQ-8C. This second sequence was chosen as screening steps from a previous study predicted the lysin would have a lower level of activity if this sequence was excluded. The resulting endolysin was tested in vivo against skin infections by *A. baumannii*. P307 alone had a significant effect against the bacterial infection but it was demonstrated that the modification of the C-terminal domain did in fact increase lytic activity log-fold. It was reported that the engineered lysin P307SQ-8C was 10–15-fold more active than its predecessor.

### 7.3. Overcoming Barriers in Drug Delviery Methods

As previously mentioned, a disadvantage to the use of endolysin therapy is the issue of drug delivery. Endolysins are readily degraded by the body’s natural defence mechanisms, and this could lead to poor bioavailability and irreversible damage to the integrity of the protein structure. As we look to the future of endolysin therapy and next-generation lysins, it is important to consider ways to overcome this hurdle to make phage-derived proteins a more favourable choice for therapeutics.

#### 7.3.1. Nanoparticles

Nanotechnology is a relatively new field of science that aims to connect the biological and psychical realms through nanostructures. Nanoparticles are usually small-sized nanospheres that are constructed at the molecular level. They are designed to be able to navigate the human body with ease and therefore are excellent candidates as peptide carriers. These spheres can be used to encapsulate a protein-based drug that would otherwise be degraded in its environment. Encapsulation of lysins could be a way forward to allow the endolysin to reach its desired target without being destroyed. It can also allow for a specific dose to be administered at the site of infection. This is essentially a targeted mode of transport for an encapsulated drug.

An example of nanotechnology being adapted to endolysin therapy is the encapsulation of the endolysin LysRODI in pH-sensitive liposomes. This 2020 study highlighted the potential for a lysin to reach its target bacteria with a timed release based on environmental cues [51]. LysRODI is a lysin specific to *S. aureus*. The lysin was encapsulated into liposomes produced by Nanovex Biotechnologies that were designed to be sensitive to pH 5.5 and below. A timed release at this pH could work for human environments such as the skin or in certain food products. A turbidity reduction assay was carried out to confirm that the lysin retained its activity after the encapsulation process. The liposomes containing LysRODI were then tested against biofilms of *S. aureus* strains. The reduction in bacterial counts as not as high as the free form of the lysin, but were still significant against the biofilm formation of all tested *S. aureus* strains.

A 2017 study also encapsulated an endolysin but the release of lysin was thermally triggered. This study targeted skin infections caused by *S. aureus.* The truncated endolysin CHAP_k_ and bacteriocin lysostaphin were encapsulated in Poly (*N*-isopropylacrylamide) nanoparticles which were designed to release at a temperature of 37 °C. This release temperature was chosen as, during infection, the temperature of the human skin rises from 32 to 37 °C, and thus the lysin and bacteriocin would only be released in the infected region. Lysostaphin and the endolysin have a synergistic effect against *S. aureus* infection which was not affected by the encapsulation process. The results of this study showed that *S. aureus* treated at 32 °C with the nanoparticles continued to grow at normal rates, while there was a significant reduction in CFU/mL at 37 °C [52].

The adaptation of nanoparticles as a vehicle for endolysin drug delivery is a novel way of making an endolysin a viable treatment option. As previously mentioned, protein-based drugs can often be vulnerable when faced with environmental challenges in the body, for example undesirable pH levels or the presence of protein-degrading enzymes. Nanoparticle encapsulation offers a novel way of protecting protein-based drugs until they reach their site of action, and may give otherwise inapt endolysins an opportunity to become an effective therapeutic.

#### 7.3.2. Lysocins

“Lysocins” are a new concept that involve the use of a bacteriocin to deliver a phage-derived lysin across the outer membrane of Gram-negative bacteria. This approach has been used to target the ESKAPE pathogen *Pseudomonas aeruginosa*. It has been previously mentioned that endolysins targeting Gram-negative species are harder to deploy due to the nature of the bacterial cell. In the parent phage, this barrier is overcome by the production of lysins inside the cell and the lysins act to lyse the cell from within. However, lysins applied exogenously are usually unable to reach the inner membrane of Gram-negative cells. In 2019, a study used “S-type pyocins” that are naturally produced by Gram-negative bacteria such as *Pseudomonas aeruginosa.* These S-type pyocins are bacteriocins encoded by bacteria that favour intraspecies competition in their environments, and so act against other Gram-negative species. In this study, the domain that allows pyocin S2 to cross the Gram-negative outer membrane was utilised. This transporter domain was fused to the *P. aeruginosa* targeting lysin GN4. The resulting “lysocin” was called PyS2-GN4. The study showed PyS2-GN4 to have a narrow spectrum of activity against *P. aeruginosa* with the FpvAI receptor [53].

### 7.4. Emphasis on Drug Discovery

In 2014, an initiative was set up by the Pew Charitable Trust to highlight the lack of products in development in antibiotic pipelines of the pharmaceutical industry [54]. This initiative was cantered around the need to develop and bring to market novel antibiotics to combat the growing threat of antibiotic resistance. Given that the vast majority of antibiotics on the market today were discovered decades ago, there is an increasing need to identify novel antimicrobials—especially those targeting multidrug-resistant strains of bacteria. Initiatives such as these could play an important part in the development and production of endolysins by pharmaceutical companies. The analysis carried out by the Pew Trust showed there are simply too few antibiotics in development to address patient needs. Reports such as theses place emphasis on the need for pharmaceutical companies to focus on the development of new antimicrobials, and as a result could focus attention on the possibility of endolysins as an alternative to antibiotics.

## 8. Current Commercial and Medical Applications of Endolysins

Although endolysin therapy is a relatively new concept, and the majority of discoveries have not gone beyond the research lab, some companies in both Europe and America have made significant strides in getting their product to the market. Even in its infancy, endolysin therapy has already begun to contribute to medicine and has some promising commercial products coming to the fore. In this review, we have discussed some early endolysin findings, the roadblocks that can be encountered along the path to commercialisation, and what we can expect to see in the future. This section will focus on the current applications of endolysins.

### 8.1. Clinical Trials

At the Rockefeller University, a biotech company called ContraFect has acquired the rights to nine phage-derived lysins. This company’s focus is the “molecular treatment of infectious disease”, with a strong focus on endolysin therapy. The lysins at this institute specifically target members of the ESKAPE pathogens including *S. aureus, Streptococcus pneumoniae, Enterococcus faecalis* and *B. anthracis*.

Exebacase (lysin CF-301) is one of ContraFects recombinant lysins that has been designed to target a range of *Streptococcus* and *Staphylococcus* species with an aim to treat infective endocarditis in humans. These species are notorious for forming biofilms on cardiac surfaces and can be difficult to target with traditional antibiotics. This particular lysin was the first of its kind to enter human clinical trials in the U.S. The lysin showed success in phase II clinical trials, where the recovery rate of MRSA-induced infective endocarditis increased by 42.8% when the lysin was administered alongside the standard antibiotic treatment for this condition [55]. This lysin is expected to enter phase III human clinical trials in the U.S. ContraFect has reported that upon meeting with the U.S. Food and Drug Administration (FDA) and the end of phase II it was indicated that the success of Exebacase in phase III clinical trials could support the Biologics License Application for the commercialisation of this endolysin product. The success of this lysin in clinical trials is so far showing great promise and could pave the way for the approval of endolysin therapy in a clinical and medical setting. 

SAL200 is another phage-derived protein that has showed success in clinical trials. A report published in 2017 summarised results of phase I clinical trials in the U.S. In this trial, it was reported that SAL200, also called N-Rephasin^®^ SAL200, is suitable to be a drug that is administered intravenously. This endolysin therapeutic is a recombinant form of the lysin SAL-1 targeting antibiotic-resistant *Staphylococcus* infections. The 2017 study was focused primarily on the pharmacokinetics of the lysin in the human body, and tolerance for the lysin when administered intravenously. In total, 34 healthy male volunteers completed the study in full. Different doses of SAL200 were administered to different volunteers in a single dose and their condition was monitored. No serious side effects were reported throughout the study, indicating a good safety profile. The endolysin drug also showed good pharmacokinetic characteristics [56]. This was the first study of its kind in the administration of an endolysin-based drug, and the findings indicate that lysin therapy could be a safe and viable therapeutic option, although the authors state further investigations should be carried out.

### 8.2. Commercial Products

Artilysin^®^ (artificial lysin) is an antimicrobial platform created by biotechnology company Lysando and AiCuris (a spin-off company of Bayer founded in 2006). This platform encompasses the use of muralytic endolysins applied exogenously which can pass through the outer membrane of Gram-negative pathogens. A lipopolysaccharide disruption protein is fused to the catalytic domain or cell wall-binding domain of the endolysin without altering its secondary and tertiary structure. This allows the Artilysin to locally penetrate the LPS outer membrane by interfering with its ionic and hydrophobic forces leading to instability of the membrane. The endolysin segment of the moiety may now pass through the outer membrane to target the peptidoglycan layer [57].

Art-175 is an Artilysin developed for the treatment of multidrug-resistant *Pseudomonas aeruginosa*. Art-175 consists of a sheep-derived peptide (SMAP-29) fused to the endolysin KZ144. Due to the success seen with this modified endolysin in vitro, the antimicrobial was considered for the treatment of otitis in dog models. Otitis externa results in chronic inflammation of the canine ear canal, often caused by *P. aeruginosa*. Two case studies on two dogs were carried out to test the ability of Artilysins to tackle usually difficult to treat infections. Antibiotics were initially used to treat both dogs, but after 3–6 weeks, no signs of improvement were seen. Upon the application of Art-175 in the first subject, the infection and inflammation of the ear was completely healed after six days, with no relapse being reported. These results were mirrored in the second dog, who showed recovery from infection eight days after the initial treatment [29].

Micreos is a company whose mission is to provide an alternative to antibiotic treatment. One of their current products on the market is Gladskin, introduced in 2013, which is a targeted endolysin therapy for *S. aureus* infections that are known to aggravate skin conditions such as eczema and acne. Gladskin is a topical cream containing the endolysin SA.100. A study published in Case Reports in Dermatology reported on the ability of Staphefeckt SA.100 to tackle chronic *S. aureus*-related dermatoses [58]. This study followed three individuals, two control groups, and one patient receiving the Gladskin treatment. All three subjects had been treated with standard antibiotics and steroids, but the infections persisted with consistent recolonisation of the area and resistance to treatment. Treatment with SA.100 resulted in the complete absence of bacterial infection within days, although stopping treatment still resulted in the patient returning to the clinic with recurring symptoms. However, the study concluded that continued use of SA.100 was capable of continuously suppressing the infection without the occurrence of resistance to the endolysin. It was also found that SA.100 had a very targeted mechanism of action and did not harm the commensal skin bacteria, unlike the traditional antibiotic solution [58]. Although SA.100 is expressed in recombinant *E. coli*, there is little chance of there being a safety issue during production. *E. coli* is a well-known Gram-negative pathogen, but the strains used for recombinant expression of proteins are usually harmless and are chosen based on their high expression rate, fast growth rate, and well-characterised genome [59].

Micreos also have another potential endolysin product in the pipeline in recent years. XZ.700 is a recombinant chimeric lysin that is targeted to drug-resistant species of *Staphylococcus aureus*. The lysin XZ.700 began phase I/IIa clinical trials in 2020 for the treatment of atopic dermatitis. XZ.700 targets the bacterium that is often responsible for flare-ups of this skin condition. This clinical trial will assess the safety of the product on a cohort of 48 patients and is expected to publish results by the end of 2021 [60].

In October of 2020, L’Oréal announced that they will be joining Micreos to expand their expertise in the skin microbiome and biotechnology field. Under this agreement, Micreos will give L’Oréal access to its endolysin research to treat skin conditions. This is a significant step forward for endolysin therapy.

Gladskin by Micreos, Exebacase, and the Artilysin platform are certainly success stories when it comes to the commercialisation of endolysin therapy. These companies have succeeded in creating effective lysins with viable applications and, more importantly, are continuing their research, advancing clinical trials and seeking future investments. It is worth noting that all of the companies mentioned have other lysins currently under development.

## 9. Conclusions

Endolysin research has certainly grown in interest, particularly over the past ten years. What started out as a decline in phage therapy due to the rise of antibiotics has been revived and is now a growing area of research that has great potential. From a clinical perspective, phage-derived proteins could become a viable treatment option for a number of pathogens, including multidrug-resistant bacteria. Not only are lysins proving to be effective antimicrobials alone, but they are also showing synergistic abilities that could give existing antibiotics a new lease of life. Despite the research in favour of endolysins as a therapeutic, they still face challenges to become a commonplace antimicrobial. Initial safety studies to date have indicated that endolysins are suitable for use in medicine but this bank of knowledge is limited, and further research must be carried to ensure phage-derived lysins can be trusted in a clinical setting. Expansion of this research will allow regulatory bodies to make more informed decisions on the commercialisation of endolysin-based products. Nonetheless, the desirable catalytic activity, potential for engineering and targeted mode of action give endolysins a strong advantage over conventional antimicrobials.

## Data Availability

Not applicable.
